# Cortical alpha activity predicts the confidence in an impending action

**DOI:** 10.3389/fnins.2015.00243

**Published:** 2015-07-28

**Authors:** Jan Kubanek, N. Jeremy Hill, Lawrence H. Snyder, Gerwin Schalk

**Affiliations:** ^1^Department of Anatomy and Neurobiology, Washington University School of MedicineSt. Louis, MO, USA; ^2^Department of Biomedical Engineering, Washington University in St. LouisSt. Louis, MO, USA; ^3^National Center for Adaptive Neurotechnologies, Wadsworth Center, New York State Department of HealthAlbany, NY, USA

**Keywords:** perceptual decision-making, certainty, neural correlates, human, EEG

## Abstract

When we make a decision, we experience a degree of confidence that our choice may lead to a desirable outcome. Recent studies in animals have probed the subjective aspects of the choice confidence using confidence-reporting tasks. These studies showed that estimates of the choice confidence substantially modulate neural activity in multiple regions of the brain. Building on these findings, we investigated the neural representation of the confidence in a choice in humans who explicitly reported the confidence in their choice. Subjects performed a perceptual decision task in which they decided between choosing a button press or a saccade while we recorded EEG activity. Following each choice, subjects indicated whether they were sure or unsure about the choice. We found that alpha activity strongly encodes a subject's confidence level in a forthcoming button press choice. The neural effect of the subjects' confidence was independent of the reaction time and independent of the sensory input modeled as a decision variable. Furthermore, the effect is not due to a general cognitive state, such as reward expectation, because the effect was specifically observed during button press choices and not during saccade choices. The neural effect of the confidence in the ensuing button press choice was strong enough that we could predict, from independent single trial neural signals, whether a subject was going to be sure or unsure of an ensuing button press choice. In sum, alpha activity in human cortex provides a window into the commitment to make a hand movement.

## 1. Introduction

The ability to make a good choice among multiple alternatives is critical to animals' survival and to human well-being. Over the past decade, systems neuroscience has begun to uncover the neural correlates of the variables that characterize subjects' decision-making (Platt and Glimcher, 1999; Shadlen and Newsome, 2001; Heekeren et al., 2004, 2008; Gold and Shadlen, 2007; Tosoni et al., 2008; Wang, 2008; Andersen and Cui, 2009; Ho et al., 2009; Kable and Glimcher, 2009; Ratcliff et al., 2009; Wunderlich et al., 2009; O'Connell et al., 2012). In these studies, researchers estimate the variable on which subjects base their decision in a given task, a “decision variable” (DV). When the proportion of choices of one of the options is plotted against the DV, one universally observes a graded and continuous (e.g., sigmoidal) relationship, a psychometric curve. These results suggest that a DV may reflect a graded commitment, or a degree of the subject's confidence, to choose a given option. In consequence, the neuronal modulations that correlate with DVs (Shadlen and Newsome, 2001; Heekeren et al., 2004, 2008; Gold and Shadlen, 2007; Wang, 2008; Andersen and Cui, 2009; Ho et al., 2009; Kable and Glimcher, 2009; Ratcliff et al., 2009; Tosoni et al., 2008; Wunderlich et al., 2009; O'Connell et al., 2012) may reflect a subject's confidence in their choice.

In a few more recent studies, researchers probed the subjective aspects associated with the degree of commitment (i.e., the choice confidence) more directly, using confidence-reporting tasks in animals (Kepecs et al., 2008; Kiani and Shadlen, 2009; Middlebrooks and Sommer, 2012; Komura et al., 2013). Electrophysiological recordings revealed that choice confidence is an important and potentially novel factor in modulating neural activity in multiple regions of the brain. Of particular interest, one of these studies (Kiani and Shadlen, 2009) found that the modulation of neuronal firing rates in lateral intraparietal area (LIP) by a DV that characterized the amount of information in a visual stimulus in many previous studies (e.g., Shadlen and Newsome, 2001; Gold and Shadlen, 2007) could in part be captured by the degree of a monkey's confidence in a choice, i.e., whether a monkey is sure or unsure to make a choice (Kiani and Shadlen, 2009). Furthermore, a regression model suggested that the modulation of LIP firing rates due to the choice confidence could not be entirely explained by the modulation of LIP firing rates due to the DV. The choice confidence correlated with LIP firing rates in a manner partially independent of the DV.

These findings demonstrate that choice confidence can be an important and possibly independent new factor in modulating neuronal activity in decision tasks. Given these findings, we tested how choice confidence modulates neural signals recorded in a perceptual decision task in humans. One of the main benefits of approaching this issue in humans is that humans are capable of accessing and explicitly reporting their confidence in a choice (McDougall, 1921; Vickers, 1979; Baranski and Petrusic, 1994; Koriat, 2011; Yeung and Summerfield, 2012; De Martino et al., 2013). While the subjects performed the task, we recorded cortical activity using electroencephalography (EEG). We designed the task to control for potential confounds. In particular, we controlled for eye movement by imposing fixation, for activity of peripheral muscles, for a particular kind of choice (hand, eye movement), and for reaction time. We found that subjects' confidence in committing to a button press is predicted by and can be inferred from parietal alpha activity.

## 2. Methods

### 2.1. Subjects

Ten right-handed human subjects participated in the study. The subjects comprised 4 females and 6 males, aged 23–72 (mean 41.1). All subjects were healthy, had normal hearing capacity, and gave informed consent through a protocol reviewed and approved by the Wadsworth Center Institutional Review Board. Subjects were paid for their participation.

### 2.2. Data collection

Subjects sat in a comfortable chair 60 cm in front of a flat-screen monitor. They wore a 16-channel EEG cap (see Section 2.7). Subjects wore headphones (MDR-V600, Sony) that presented a stereo auditory stimulus (see Section 2.4). The right arm rested comfortably on a pillow that was placed on a fixed table. The subjects' right hand was steadily holding a joystick (ATK 3, Logitech); subjects were ready to simultaneously press the front and top buttons of the joystick using their right index finger and the right thumb, respectively. Gaze position of each eye was measured using an eye tracker (Tobii T60, Tobii Technology, Inc., Sweden) that was integrated into the flat-screen monitor. Acquisition of EEG signals, eye gaze parameters, joystick button press parameters, as well as control of the experimental design were accomplished with the BCI2000 (RRID:nif-0000-00251) system (Schalk et al., 2004).

### 2.3. Task

The task is a variant of that used previously (Kubanek et al., 2013). In comparison to that previous task, the present task incorporates the confidence reporting period, and eliminates the variable delay period. As in the previous task, subjects had to fixate prior to a response, which effectively eliminates a possible eye movements confound. In the present task, we in addition controlled for the possible early movements of the hand or the body by measuring the EMG activity of the forearm muscles.

**Figure 1:**
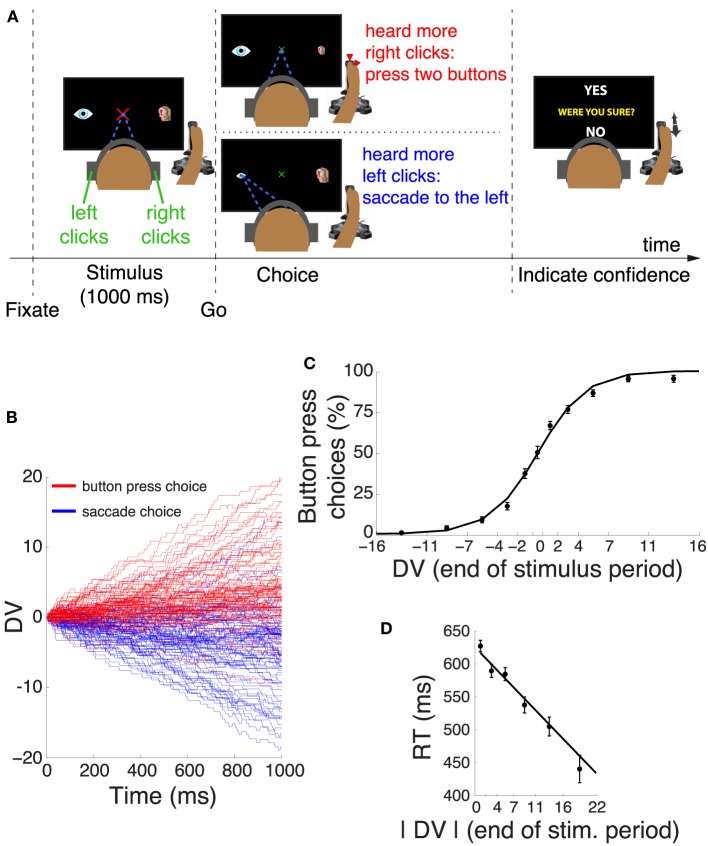
**Decision task and choice behavior**. **(A)** After acquiring a fixation cross, subjects listened to a binaurally presented auditory stimulus. Subjects decided whether they heard more click sounds in the right ear or in the left ear. If subjects heard more clicks in the right ear, they pressed two buttons of the joystick with their right index finger and the thumb. Otherwise, they made a saccade to the eye icon on the left side of the screen. After the choice, subjects indicated whether they were sure or unsure about the choice by moving the joystick to YES or NO tags, respectively. One of these tags randomly appeared in the upper part and the other in the lower part of the screen. **(B)** Temporal progression of the decision variable (DV) on each trial during the stimulus interval, separately for trials that resulted in a button press (red) and trials that resulted in a saccade (blue). For clarity, the figure shows 100 randomly selected trials for each choice. **(C)** Mean ± s.e.m proportion of button press choices as a function of the binned DV at the end of the stimulus period. The individual data points were fitted with a logistic function. **(D)** Mean ± s.e.m reaction time as a function of the absolute value of the DV at the end of the stimulus period. The individual data points were fitted with a line.

Each trial (Figure 1A) began with the presentation of a red fixation cross, subtending 2° of visual angle. Subjects had to fixate at the center of the cross, and keep the eye gaze within a radius of 2°. An absence of eye gaze within the fixation radius for more than 150 ms was considered as a break of fixation. After acquiring fixation, two icons appeared, 15° to the right and 15° to the left of the fixation cross. The right icon was a sketch of a joystick with highlighted top and front red buttons. The left icon was a sketch of the eye. At the same time, subjects were presented with a stereo auditory stimulus (click sounds, see Section 2.4), 1.0 s in duration. Subjects had to determine whether they heard more clicks in the right ear or more clicks in the left ear. After the stimulus, the fixation cross shrank to 1° in diameter and changed its color to green. This event cued the subjects to make a movement (choice). If subjects heard more clicks in the right ear than in the left ear, they simultaneously pressed the front and the top button of the joystick using the right index finger and the right thumb, respectively. In the analyses, movement onset was taken as the time of the earlier button press. On the other hand, if subjects heard more clicks in the left ear than in the right ear, they made an eye movement to the left icon. A successful choice was communicated to the subject by shrinking the icon corresponding to the chosen movement (the eye icon or the joystick icon) from 2° in size to 1° in size. If subjects broke fixation or pressed any button before or within 200 ms after the appearance of the go cue, or if they failed to indicate a response within 800 ms after the go cue, the trial was aborted and excluded from the analyses. A trial was also aborted if subjects responded with both movements, or if subjects made a saccade to the right icon. The type of error was indicated to the subjects in red, large-font text (“TOO EARLY,” “TOO LATE,” “MOVED BOTH”).

After making a choice, a text “WERE YOU SURE?” was displayed in the center of the screen, along with “YES” and “NO” tags in the periphery. The location of these two tags was randomized on each trial. Specifically, one of these tags randomly appeared in the upper part of the screen and the other tag appeared in the lower part of the screen. Subjects were instructed to move the joystick in the direction of the appropriate tag and to press the two buttons of the joystick to confirm their selection. If subjects did not select a tag within 5 s, the trial was aborted and discarded.

**Figure 2:**
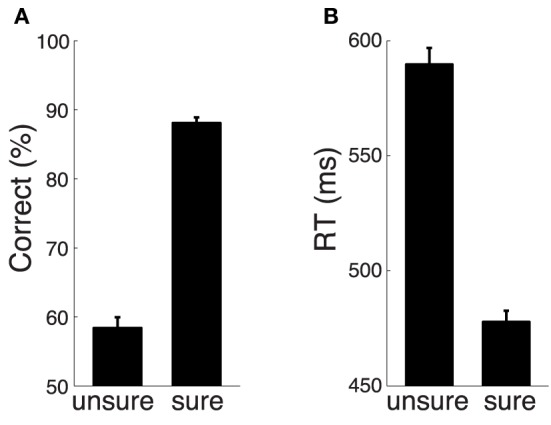
**The choice confidence reflected in behavior**. **(A)** Mean ± SEM proportion of subjects' correct choices as a function of subjects' confidence. **(B)** Mean ± SEM reaction time as a function of subjects' confidence.

After subjects indicated their confidence, they were given feedback, 0.6 s in duration, which was a string indicating a particular number of points followed by the “c” symbol. A correct choice entailed a gain of 10 points. An incorrect choice incurred a loss of 10 points. Furthermore, when the online algorithm (see below) estimated that a decision was difficult (−0.5 < *E* < 0.5) and subjects indicated NO, or if a decision was estimated to be easy (*E* < −0.5 or *E* > 0.5) and subjects indicated YES, additional 10 points were added to the sum; otherwise, 10 points were subtracted. The offset of feedback was followed by a variable inter-trial interval, 0.6–1.2 s in duration. The feedback was shown to alleviate the possible argument that subjects had little incentive to accurately report their level of confidence, compared to studies in animals in which animals were specifically rewarded for reporting particular levels of confidence (Kiani and Shadlen, 2009). Nevertheless, since the subjects were only told that they were paid for their participation (they were not paid for and no statement was made in regard to the payment for their performance), they may have ignored the feedback points, and so the confidence reports could still be sub-optimal. However, the objective measures of the confidence reports (Figure 2) suggest that the confidence reports properly reflected the amount of information in the stimulus. Notably, our neural effects cannot be explained by a subject's anticipation of a particular feedback outcome, because there was no effect during saccade choices (**Figure 7**).

Subjects made a valid choice in 85.1±3.0% (mean ± SEM across the 10 subjects) of trials. The error rate for the too late responses was 5.0±1.5%, for the too early responses 6.6±2.1%, for the responses with both effectors 0.5±0.2%, and for making a saccade to the wrong (rightward) icon 2.7±0.9%. Each of the 10 subjects completed at least two sessions of 100 valid trials each. Two subjects completed an additional session of 50 valid trials, and five subjects an additional session of 100 valid trials.

### 2.4. Auditory stimulus

The stimuli used in this study are identical to those used previously (Kubanek et al., 2013). Briefly, the auditory stimulus presented to each ear consisted of a train of brief (0.2 ms) click sounds drawn from a homogeneous Poisson process. Each train lasted 1.0 s. The stereo stimulus was composed such that the sum of clicks presented to the left ear (*C*_*l*_) plus the sum of clicks presented to the right ear (*C*_*r*_) summed to one of four fixed values *C*_*l*_ + *C*_*r*_ = Ω, Ω ∈ {25, 32, 39, 46}. The value of Ω was drawn randomly on each trial.

### 2.5. Online adaptive algorithm

An online algorithm ensured that subjects were often confident and often not confident about their decision in different trials. To achieve that, prior to the start of each trial, we randomly drew a number *E* from a uniform distribution over the interval (−1, +1). The program then randomly selected one of the 10 pre-generated auditory stimuli with *C*_*r*_ and *C*_*l*_ that—according to the current model—most closely correspond to the generated *E*. Using the same online adaptive model as in a previous study (Kubanek et al., 2013):

$$\begin{array}{l} E=\frac{2}{1+\exp\left(-\beta \frac{C_{r}-C_{l}}{C_{r}+C_{l}}\right)}-1, \end{array}$$

it follows that

$$\begin{array}{l} C_{r}=\frac{1}{\beta }\ln\left(\frac{\left(E+1\right)∕2}{1-\left(E+1\right)∕2}\right)\Omega , \end{array}$$

where Ω = *C*_*r*_ + *C*_*l*_ and thus *C*_*l*_ = Ω − *C*_*r*_.

The difficulty of the presented stimuli was adjusted to the performance of each subject. Our objective was to keep each subject at 75% of correct responses. To achieve this, the program adapted the value of β (initial value β = 8) to each subject's performance over the last 20 trials according to the following update rule:

$$\begin{array}{l} \beta_{new}=\beta_{old}1.2^{\left(A-75\right)∕10} \end{array}$$

where *A* is the accuracy, in %, over the past 20 trials. This procedure allowed subjects to perform close to the desired accuracy (76.6±1.2% (mean ± SEM) over the 10 subjects).

### 2.6. Decision variable

To describe the choice behavior of subjects in this task, we devised a decision variable (DV) according to signal detection theory (Gold and Shadlen, 2007). In particular, a simple DV constructed from discrete, independent pieces of evidence (click sounds) can be expressed in terms of the logarithm of the likelihood ratio (logLR):

$$\begin{array}{l} \text{DV}\left(t\right)=\log LR\left(t\right)=\overset{i\left(t\right)}{\underset{i=1}{\sum }}\log\frac{P\left(e_{i}\;|\;\text{button}\right)}{P\left(e_{i}\;|\;\text{saccade}\right)}, \end{array}$$

where the sum runs from the first click up to the last click *i*(*t*) occurring prior to or at time *t*, *e*_*i*_ is the *i*-th click (rightclick or leftclick), and *P*(*rightclick*|*button*) is the probability that a click is a right click given a button press choice (and analogously for the 3 other combinations of the arguments of *P*). These probabilities were computed from the frequencies of the summed right (or left) clicks over all trials of a given choice.

This DV captured both the subjects' choice behavior and the reaction time (Figures 1B–D). Our results did not change when we considered a different definition of the DV (Kubanek et al., 2013). Both forms of DV predicted subjects' choice behavior similarly well, and both produced similar neural effects. We here used the formalism of the sequential analysis of the signal detection theory (Gold and Shadlen, 2002, 2007).

### 2.7. Electrophysiological recordings

As in a previous study (Kubanek et al., 2013), neural data were recorded using a 16-channel EEG cap (Electro-Cap International, Inc., Eaton, OH). The channels were positioned according to the International 10–20 method of electrode placement (F3, Fz, F4, T7, C3, Cz, C4, T8, CP3, CP4, P3, Pz, P4, PO7, PO8, Oz). The left and right mastoids served as ground and reference, respectively. The signals were re-referenced to a common average reference (CAR): for a given channel, the voltage waveform resulting from averaging the voltage waveforms over all channels was subtracted from the voltage waveform at that given channel (Kubanek et al., 2013). The neural signals were anti-alias-filtered and acquired with a g.USBamp series B amplifier (g.tec, Austria) at 24-bit resolution at a rate of 256 Hz.

The lack of an effect during saccades presents a powerful negative control against a possible general artifact. Nonetheless, we additionally tested the effects of an artifact removal procedure on our results (Murray et al., 2008). The procedure first removes low frequency trends in the raw EEG signals using a high-pass filter (we used an IIR high-pass with a cutoff frequency of 0.05 Hz). Subsequently, the procedure removes all trials in which during the stimulus period the EEG signals at *any* channel exceed +100μV or fall below −100μV. This procedure removed 14.1% of trials. This procedure had minimal impact on the results (see Section 3). Lowering the exclusion criterion yet further, to 75μV, removed 30.3% of trials, and again led to the same principal effects (not shown).

### 2.8. Measurement of hand EMG

In this task, we recorded, besides electroencephalographic (EEG) activity of the cortex, also the electromyographic (EMG) activity of anterior forearm muscles. Bipolar measurements were made through two surface leads (GS27 pre-gelled disposable sEMG sensors) placed 2 cm apart along the flexor carpi radialis, and two surface leads placed 2 cm apart along the flexor digitorum superficialis (which in part potentially also reflects activity of the palmaris longus). For both muscles, the lead further apart from the wrist served as the reference. The EMG signals were acquired using an additional g.USBamp series B amplifier (g.tec, Austria) at 24-bit resolution at a rate of 256 Hz. The EMG signals were filtered in a typical frequency range in which EMG is observed using an IIR band-pass filter (20–100 Hz, 80 dB cut-off at 19 and 105 Hz). Using a lower cutoff, of 5 Hz, had only minimal impact on the EMG effects. The EMG power was evaluated in the same time windows as the EEG power. The two muscles showed similar effects. We report the activity of the more sensitive flexor carpi radialis.

We defined the time at which two traces start to significantly deviate (**Figures 5B,C**) by finding the first time sample for which a Wilcoxon rank sum test (we used this test because the compared conditions are independent, with different numbers of trials) returns a *p*-value lower than 0.01, and this condition holds for at least 10 consecutive time samples.

### 2.9. Time-frequency analysis of the neural data

**Figure 3:**
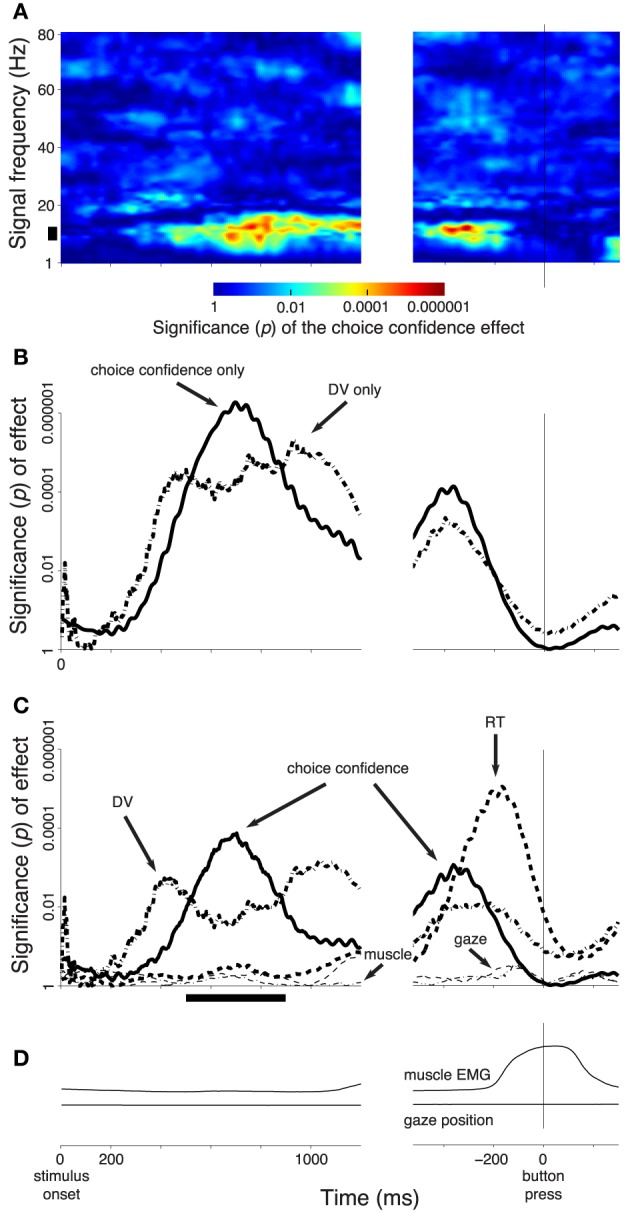
**Subjects' confidence in a button press choice reflected in cortical signals**. In all panels, effects are shown as a function of time throughout the trial. Data are aligned on the stimulus onset (left), and on the time of the button press (right). The figure shows effects during all button press choices. **(A)** The significance of the weight (*p*-value associated with the *t*-statistic, see color bar) of the choice confidence regressed on the neural activity (i.e., the contrast between sure and unsure trials), at each time throughout the trial and at each frequency in the range from 1 to 80 Hz. The thick vertical bar spanning the range 8–12 Hz denotes the alpha band. **(B)** The significance of the weight (*p*-value associated with the *t*-statistic) of the choice confidence (solid) or the DV (dash-dot), separately regressed on the neural signals measured in the alpha band, as a function of time. **(C)** The significance of weight of individual factors in a multiple regression on the neural signals measured in the alpha band, as a function of time. The factors include the choice confidence, the DV, the reaction time (RT), the horizontal eye position, and the EMG activity of the forehand muscle flexor carpi radialis. The solid bar below the abscissa denotes the period of significance of the choice confidence effect during the stimulus period. **(D)** The mean activity of the forehand muscle flexor carpi radialis and the mean horizontal eye gaze position. In all panels, signal power was computed in 300 ms sliding windows (30 ms overlap) and averaged over all channels. The behavioral traces in **(D)** were computed as means over the corresponding 300 ms windows.

In this analysis (Figure 3A), the neural signals were evaluated in 300 ms windows sliding through the trial in 30 ms steps. In each window, we estimated the power spectral density for each frequency in the range from 1 to 80 Hz using an autoregressive (AR) model of the order 15, applying Burg's method (function pburg in Matlab). The choice of the AR order did not significantly influence the results of Figure 3A. The analysis window was chosen to be wide enough to provide reliable estimates of the power spectral density for low frequencies (e.g., for a 4 Hz signal, the 300 ms window spans at least one full period), and short enough to capture the temporal variability in the neural signals. For increased temporal resolution, in **Figures 5B,C**, we used a shorter window of 100 ms.

### 2.10. Computation of the power in the alpha band

We computed the power in the alpha band by first filtering the signals of each EEG channel in the alpha band (8–12 Hz; 80 dB cutoffs at 7 and 13 Hz) using an IIR filter. To avoid phase distortion, we applied the filter using the filtfilt function in Matlab (The MathWorks, Natick, MA). This filtering method performed zero-phase digital filtering by processing the input data in both the forward and reverse directions.

We then computed the power of the filtered signals in 300 ms windows sliding through the trial in 30 ms steps. For increased temporal resolution, the alpha power in **Figure 5B** was computed in shorter, 100 ms windows, and is in this figure visualized as a relative decrease in power (“modulation”) with respect to the mean power for a given subject (Kubanek et al., 2013). Thus, the higher the modulation, the more desynchronized the neural signals in the alpha band.

### 2.11. Linear models

In Figure 3A, we investigated the neural representation of the choice confidence in a multiple regression on the neural activity on each trial:

(1) $$\begin{array}{l} \text{Neural}=\alpha_{1}\text{Confidence}+\overset{10}{\underset{i=1}{\sum }}\beta_{i}, \end{array}$$

where Confidence takes one of two values [sure (value of 1), unsure (value of 0)], and β_*i*_ are the intercept terms for the individual subjects. We included these additional terms in all analyses that present the significance of an effect, to make sure that the effects we report are not due to cross-subject variability. Similar effects of the choice confidence were obtained when these terms were not included in the regression.

In Figure 3A the regression was performed separately for signals at each time and frequency. Therefore, in that figure, Neural represents the power at a given time window and frequency, for a given trial. In Figures 3B,C, **7**, the neural signals were filtered in the alpha band (see above), and so in those analyses Neural represents the alpha power at each time window, for a given trial.

In Figure 3 the signal power was averaged, for a given time and frequency, over all channels. In **Figures 7, 8**, the alpha power was also averaged over all channels.

In Figures 3C, **7**, we investigated the specific representation of the choice confidence by accounting for the decision variable (DV), the reaction time (RT), the horizontal eye gaze position, the EMG activity of the forearm muscle flexor carpi radialis, and each subject in a multiple regression on the neural signal on each trial:

(2) $$\begin{array}{rcl} \text{Neural} & = & \alpha_{1}\text{Confidence}+\alpha_{2}\text{DV}+\alpha_{3}\text{RT}+\alpha_{4}\text{handEMG}\\ +\alpha_{5}\text{eyepos}+\overset{10}{\underset{i=1}{\sum }}\beta_{i}, \end{array}$$

where, in addition to Equation (1), DV is the instantaneous value of the DV (see above), RT is the time elapsed from the Go cue until a behavioral response, *handEMG* is the instantaneous EMG activity of the flexor carpi radialis muscle (see above), and *eyepos* is the instantaneous value of the horizontal eye position averaged over both eyes.

Following a regression fit (Equation 1 or 2), we evaluated the significance of the weight of the choice confidence (Figure 3A) and of the other factors (Figures 3B, **7**) and report the *p*-value of the associated t-statistic.

In **Figure 10**, we test the effects of the individual variables on the choice confidence in the following linear model:

(3) $$\begin{array}{rcl} \text{Confidence} & = & \alpha_{1}\text{Neural}+\alpha_{2}\text{DV}+\alpha_{3}\text{RT}+\alpha_{4}\text{handEMG}\\ +\alpha_{5}\text{eyepos}+\text{RandomEffectsDueToSubject}, \end{array}$$

where RandomEffectsDueToSubject are 60 additional random effect terms corresponding to grouping of each variable (5 plus intercept) by each subject (10 levels). The other terms are as defined previously, with their value computed as an average over the period of the significance of the effect of confidence (bar below the abscissa in Figure 3C). This averaging over a relatively long time period during the stimulus interval is not critical; we performed it to avoid an arbitrary selection of a particular time. Very similar results are obtained when a particular time within this broad window is selected for this analysis. Each variable was normalized between its 2.5th percentile (value 0) and 97.5th percentile (value 1). This scaling was performed to enable a fair comparison of the corresponding weights.

### 2.12. Visualization of topographies

We visualized data at each channel (**Figure 5A**) using the topoplot function available at http://sccn.ucsd.edu/eeglab/allfunctions/topoplot.html.

### 2.13. Prediction of confidence

For all training set trials (see Section 2.14 for details) of a given subject, we averaged the alpha power over all channels in the period of the significance of the effect of confidence (thick horizontal bar in Figure 3B). These neural values form two distributions, one for sure and one for unsure choices (**Figure 8A**). A classification criterion is then set at a particular point between the medians of the two distributions. That point is chosen by the experimenter according to the experimenter's demands on the level of type I and type II error (**Figure 8B**), that is, the rates of misclassifying the sure choices as unsure, and reversely. Using this scheme, a new, independent signal value of a test set (see Section 2.14) is then compared to the classification criterion. The scheme predicts that a subject is going to be sure (unsure) to choose the button press if the value is higher (lower) than the criterion.

### 2.14. Accuracy assessment

A correct prediction of choice confidence from the neural signal occurs when the scheme's confidence prediction from the neural signal (sure, unsure) matches the confidence indicated by the subject (sure, unsure). We assessed the accuracy of the prediction scheme using the leave-one-out procedure. In this procedure, trials (total number *N*) are divided into *N* − 1 training trials and 1 test trial. This division is repeated *N* times such that each time the 1 test trial is different than the previously tested trials. The scheme is trained on each of the training sets, and the accuracy is taken as the average correct classification of the test set trials.

Validating the predictions using new test set (independent of the training set) signals ensures that the same level of prediction accuracy could be achieved in real time settings.

### 2.15. Randomization analysis

We performed a permutation test to evaluate the significance of the prediction accuracy values in each subjects and to further ensure that these values reflect a relationship between the neural signals and the choice confidence. Specifically, we distorted this relationship by randomly reassigning, within each subject, the sure and unsure labels across the trials. We performed this procedure 10 thousand times. In each case, we computed the prediction accuracy in the same way as using the original data. Within each subject, we fit the distribution of the ten thousand accuracy values with the normal distribution, and used this normal distribution to assess whether the accuracy on the original data is higher than would be expected by chance (*p* < 0.05). The accuracy values that are according to this procedure significant are marked as filled blue bars in **Figure 8C**.

The individual accuracy values given by the randomization procedure were averaged together. The obtained average accuracy values in each subjects are shown as magenta bars in **Figure 8C**. Following this randomization procedure, the average accuracy fell to chance level, 50% on average across the subjects (range 49.9–50.1%). The same result is obtained when only the test set labels are randomized, i.e., when each test set label is randomly drawn from the pool of sure and unsure labels for a given subject.

## 3. Results

Humans performed a perceptual decision task in which they decided on the polarity of a stereo auditory stimulus (Kubanek et al., 2013). Subjects had to fixate a central target. When subjects heard more click sounds in the right ear compared to the left ear, they pressed a button. Otherwise, they made an eye movement (Figure 1A). Critically, following a choice, subjects indicated whether they were sure or unsure about their choice (Figure 1A). Confidence was reported using a joystick movement whose direction was randomized on each trial, and so there was no relationship between subjects' choices and confidence reports.

We described the subjects' choice behavior in this task using a decision variable (DV), according to sequential analysis of signal detection theory (Section 2). This DV represents an integral over time of the individual quanta of evidence supporting a given choice alternative. The time course of the DV on trials that resulted in a button press (red) and trials that resulted in a saccade (blue) is shown in Figure 1B. The figure reveals that the subjects' behavior followed the instruction—subjects typically selected the button press when the DV was positive (subjects heard more clicks in the right ear), and typically selected the saccade when the DV was negative (subjects heard more clicks in the left ear). We quantified this behavior by binning the value of the DV at the end of the stimulus interval and counting the proportion of choices of the button press in each bin. The result is shown in Figure 1C and confirms the impression of Figure 1B. A logistic regression that regressed the continuous DV on the subjects' binary choice revealed that the DV is a significant factor in determining the subjects' choices (significance of weight of DV in this model, *p* < 0.0001, *n* = 3104). Subjects made both choices in equal proportion (51.2% of button press choices, not significantly different from 50%, *p* = 0.22, proportion test). There was no influence of a previous choice on the current choice; the choice proportion was not affected by whether a subject chose a button press (*p* = 0.73, proportion test) or a saccade (*p* = 0.46) on the previous trial. The subjects' RT was a function of the absolute value of the DV at the end of the stimulus period (Figure 1D). The higher the absolute value of the DV, the faster the subjects responded (slope of line fit, *p* < 0.0001).

The amount of information in the stimulus was set such that each subject made easy and difficult decisions (Section 2). Consequently, subjects were often sure and often unsure about their decision. The proportion of trials in which subjects were sure across the 10 subjects was 61.4±12.9% (mean ± s.d.). In line with previous behavioral studies (Vickers, 1979; Baranski and Petrusic, 1998; Koriat, 2011), the level of the subjects' confidence was strongly reflected in decision accuracy (Figure 2A). Specifically, when subjects were sure, they were correct in 90.0% of trials. In contrast, when they were unsure, they were correct in only 62.8% of trials (chance was 50%). This effect was significant (Wilcoxon rank sum test, *p* < 0.0001). There was an influence of a previous confidence report on the current report. When subjects indicated that they were sure in the previous trial, the proportion of sure reports increased from the default 61.4% to 70.2%, and the increase was significant (*p* < 0.001, proportion test). When subjects indicated that they were unsure in the previous trial, the proportion of sure reports decreased from the default 61.4% to 51.2%, and this decrease was significant (*p* < 0.001, proportion test). In accord with previous studies (Vickers and Packer, 1982; Baranski and Petrusic, 1998), the level of confidence was strongly reflected in subjects' reaction time (RT; Figure 2B). In particular, when subjects were sure, they reacted faster (mean RT 478 ms) than when they were unsure (mean RT 590 ms). This effect was significant (Wilcoxon rank sum test, *p* < 0.0001).

We tested how the subjects' confidence level is represented in neural signals recorded using electroencephalography (EEG). A neural representation of the confidence in a choice could be confounded by the choice a subject is going to make. This is because subjects are more likely to choose the option for which they obtained more evidence, and consequently about which they are more confident, than the option for which they obtained less evidence, and about which they are less confident (Vickers, 1979). A neural effect of choice, even if binary (Donner et al., 2009) would, in this way, lead to artificial grading that would be falsely attributed to the level of choice confidence. We therefore fixed the effect of choice by specifically investigating trials that resulted in a button press (*n* = 1269 trials). We then regressed the choice confidence, along with terms representing each subject, on neural features averaged over all channels (Section 2), and report the significance of the weight of the choice confidence in this regression. We performed this regression at each time throughout each button press trial and for the power of the neural signals at frequencies ranging from 1 to 80 Hz, in 1 Hz steps (Section 2).

We report the significance of the weights (the *p*-values) instead of the weight magnitude. This is because the individual factors have different magnitudes and so the associated weights cannot be readily compared. In contrast, the weight significances are directly comparable.

Figure 3A shows the effect of the choice confidence, averaged over all channels, at each time and for each tested frequency of the neural signals. This analysis reveals that the EEG signals prominently reflect the subject's confidence level in choosing the button press. The effect is particularly pronounced in the frequency range from about 8 to about 12 Hz. This frequency range aligns with the traditional definition of the alpha band (Berger, 1969; Pfurtscheller et al., 1996). Thus, we filtered the neural signals in the alpha band and computed the power of the resulting signals (Section 2). We then applied the same regression model, again separately at each time throughout the trial.

Figure 3B (solid line) shows the significance of the weight of the choice confidence in this regression as a function of time. The figure reveals that the encoding of choice confidence by the alpha power reaches prominence at about 400 ms following the stimulus onset. The effect progressively increases and reaches its maximum at 699 ms following stimulus onset. The effect subsequently declines before reaching another, independent peak of significance briefly prior to a button press.

We next investigated the effects of the DV. To do so, we substituted the choice confidence by the DV as a factor in the regression, *ceteris paribus*. The significance of the effect of the value of the DV at each time throughout the trial (Figure 1B) on the instantaneous value of the alpha power is shown as the dash-dot line in Figure 3B. The figure reveals that the effect of the DV emerges somewhat earlier than the effect of choice confidence. The effect reaches a plateau of significance starting at about 400 ms following stimulus onset, and begins to decline after reaching the significance peak at 934 ms following stimulus onset—markedly later than the effect of the choice confidence. The effect vanishes briefly prior to a button press.

We investigated the degree to which the choice confidence and the DV exert independent leverage on the neural signal. To do so, we included the DV and the choice confidence as separate factors in the multiple regression (Section 2, Equation 2). In this extended regression, we further controlled for the reaction time (RT), the horizontal position of the eye gaze, and the activity of forearm muscles (Section 2), by including these factors as additional regressors on the neural activity in each trial. The high temporal resolution of EEG allowed us to investigate the contribution of the instantaneous value of each of these variables to the neural variability at each time throughout the decision process.

We found that the choice confidence had an effect on the neural signal that was partially independent of the effect of the DV (Figure 3C). As in Figure 3B, the effect of the DV becomes significant before the effect of the confidence. The effect of the DV begins to decrease after reaching a peak at 422 ms, while at about this time the effect of confidence starts taking over the modulatory role. As in Figure 3B, when the data are aligned to the button press, the choice confidence shows a second distinctive peak of significance.

Notably, when the data are aligned to the button press, the neural signal shows an additional prominent effect—a modulation due to the subject's RT. This effect reaches a peak 166 ms prior to a button press, and vanishes at about the time of the button press. The finding that neural activity shortly prior to a movement scales with how long it took a subject to produce the movement merits separate investigation (Tzagarakis et al., 2010; O'Connell et al., 2012).

**Figure 4:**
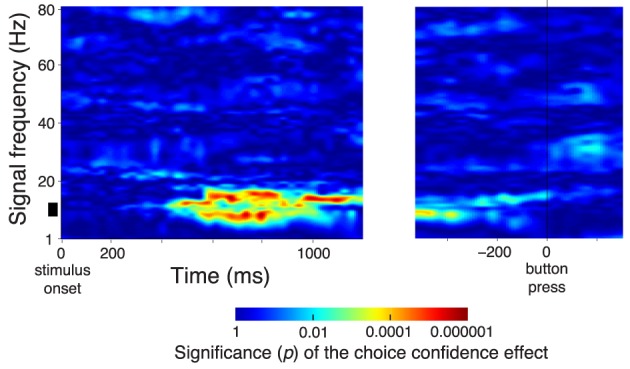
**The spectrum of the confidence-related neural effect at a single parietal channel CP3**. Same format as in Figure 3A, for data at a single parietal channel CP3.

**Figure 5:**
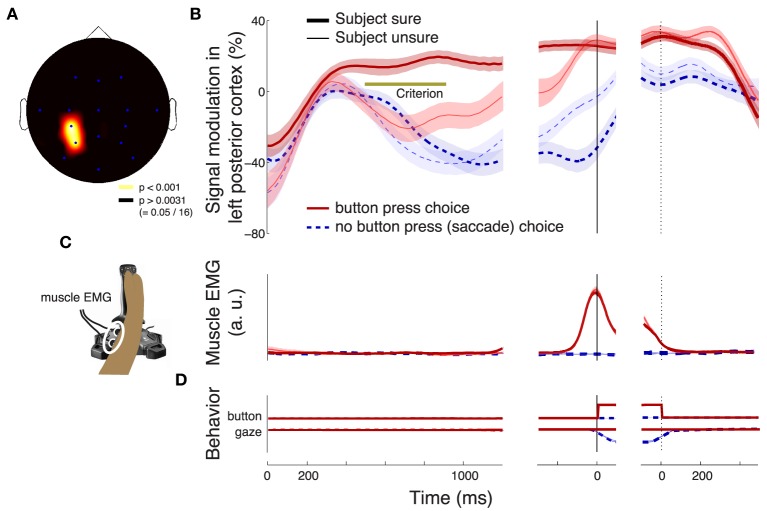
**Characterization of the neural effect of the choice confidence**. **(A)** A rendering of the regions that significantly encode the weight associated with the choice confidence in the multiple regression on the alpha-band neural signal, during all button press choices. The neural signal was averaged over the period of significance of the confidence effect (solid bar below the abscissa in Figure 3C). Regions surrounding channels with significant confidence weights (Bonferroni-corrected, *p* < 0.05∕16) are visualized in hot colors (see inset). The effect is predominantly observed at left posterior channels P3 and CP3. **(B)** Mean ± SEM alpha desynchronization in left posterior regions (averaged over channels P3 and CP3), as a function of time. Data are shown separately for sure (thick) and unsure (thin) choices, and for button press (red) and saccade choices (blue). The yellow horizontal line labeled with “Criterion” illustrates the signal threshold used to predict the confidence level in **Figure 8**. **(C)** Mean ± SEM EMG activity of the flexor carpi radialis muscle of the right hand. **(D)** Mean ± SEM button press and eye gaze signals. In **B**–**D**, data are aligned on the onset of the stimulus (left vertical line), the time of movement onset (middle vertical line), and the time of movement cessation (right dotted vertical line). In **B** and **C**, the signal power was evaluated in 100 ms windows, overlapping by 1 sample (3.9 ms).

In Figure 3, we averaged the neural features in particular frequency bands over all channels. This molar analysis approach guards against multiple comparisons (i.e., comparing multiple different sets of channels at multiple frequencies). Nonetheless, the averaging over all channels was not crucial; similar results were obtained when we chose a parietal subset of electrodes (e.g, left posterior electrodes **Figure 5A**). We further repeated the analysis in Figure 3A for a single parietal channel, channel CP3 (Figure 4). The figure demonstrates similar effects of the choice confidence over time and frequency as those shown in Figure 3A.

The effect of the confidence in a button press choice, evaluated during the period of its significance (solid bar below the abscissa in Figure 3C) is particularly pronounced in left posterior regions (channels P3 and CP3, Figure 5A). The effect is to a reduced degree present also at other channels. However, only the left posterior regions (Figure 5A) survive the Bonferroni correction for the number of channels. This localization to the left hemisphere may be expected given that subjects responded with the contralateral right hand (Donner et al., 2009). Figure 5B reveals the dynamics of the alpha band signal in the left posterior regions. The signal shows an initial activation (Kubanek et al., 2013). The signal starts to distinguish (*p* < 0.01, Section 2) the cases in which a subject is sure (dark red) and in which a subject is unsure (light red) to choose the button press at 496 ms following stimulus onset. When the subject is not sure to choose the button press (light red), the signal overlaps with the signal that represents a sure choice of the saccade (dark blue), until 828 ms following stimulus onset. Subsequently, the light red trace ramps up and reaches a level similar to the sure choices (dark red) at the time of the button press (solid vertical line). Notably, the convergence to a common level for button press choices regardless of the condition has been observed previously (O'Connell et al., 2012; Kubanek et al., 2013). This neural effect may manifest the bound that is a crucial property of the diffusion-to-bound models of decision-making in reaction-time choice tasks (Stone, 1960; Edwards, 1965; Vickers, 1970; Gold and Shadlen, 2007).

Compared to the neural signal, the electromyographic (EMG) activity of the right forearm muscle flexor carpi radialis is low and steady throughout the stimulus period (Figure 5C). The muscle activity becomes prominent only shortly prior to the button press. The muscle activity starts to significantly distinguish (*p* < 0.01, Section 2) between the two levels of confidence in a button press choice at 1020 ms following stimulus onset, although this effect likely reflects the fact that more confident choices lead to faster responses and so to a faster EMG onset relative to stimulus onset.

**Figure 6:**
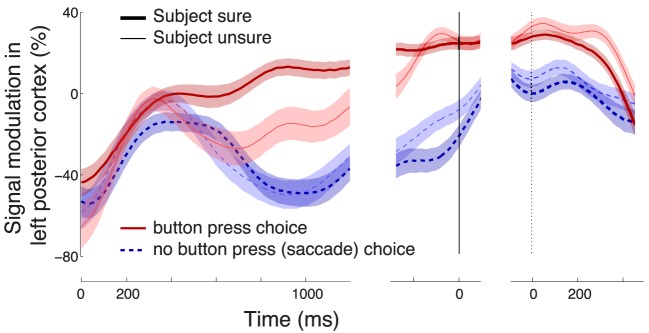
**Effects following a trial elimination procedure**. Elimination of trials in which EEG signals at any channel exceeded a particular threshold (Section 2) had only minimal impact on the confidence-related effect. Same format as in Figure 5B.

Although subjects were asked not to move during their performance, we imposed additional measures to eliminate possible movement-related confounds. In particular, subjects had to fixate a central target; trials in which subjects broke fixation were discarded. Furthermore, we measured the EMG activity of hand muscles, and included the EMG activity as a separate factor to account for a hand or body movement (Equation 2). In addition to these measures, we discarded any trial in which the EEG signals at any channel reached specific bounds (see Section 2). This procedure eliminated 14.1% of all trials. This trial-elimination procedure had only minimal effect on the results (Figure 6). Consequently, we only discarded data in which subjects broke fixation or responded too soon or too late (Section 2).

**Figure 7:**
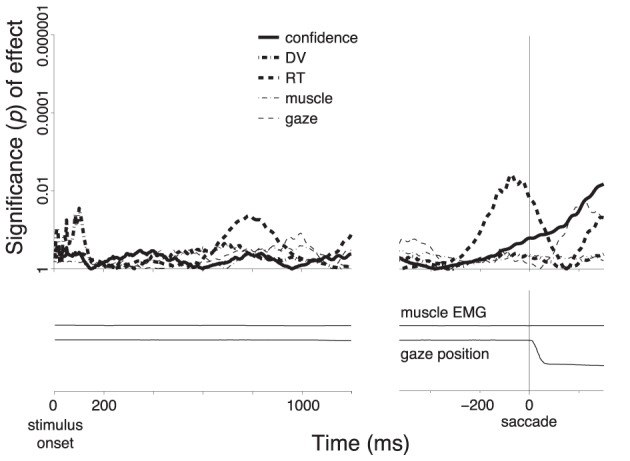
**Encoding of the individual factors during saccade choices**. Same procedure and format as in Figure 3C, for all saccade choices.

Choices that resulted in a saccade (*n* = 1331) did not show a substantial distinction between subjects' sure and unsure states (Figure 5B, blue). A distinction appears to be observed first briefly prior to the saccade (solid vertical line). However, this effect does not survive the inclusion of the controlling factors in the multiple regression. In fact, the regression reveals that this effect is mainly due to the RT (Figure 7). The specificity of the encoding of the choice confidence to button press choices suggests that the underlying neural process that is reflected in the alpha power specifically evolves in circuits pertaining to the somatomotor system.

**Figure 8:**
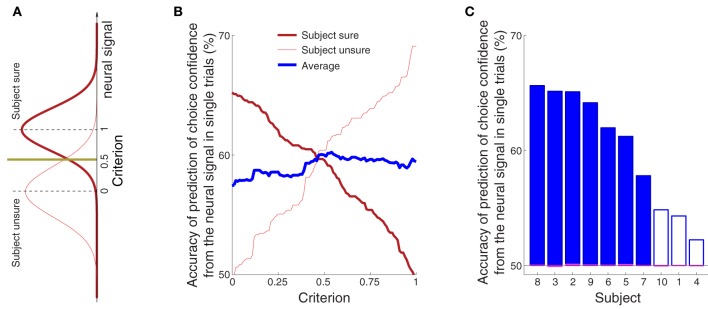
**The confidence in a forthcoming button press choice predicted from the neural signal in single trials**. **(A)** The sure and unsure choices of a button press are associated with sure and unsure distributions of values of the neural signal. A new, independent value of the neural signal is compared to a criterion value. The criterion is set by the experimenter and takes a value between 0 (value equal to the median of the unsure distribution) and 1 (value equal to the median of the sure distribution). The scheme predicts that a subject is going to be sure (unsure) if the neural value is higher (lower) than the criterion. **(B)** Average prediction accuracy as a function of the criterion, separately for sure (thick red) and unsure (think red) choices, and their average (thick blue). If the criterion is moved to a lower value, such that it accounts for a larger portion of the sure distribution in **(A)**, the ability to predict that a subject was sure will be higher (thick red), and the ability to predict unsure will be close to chance (thin red). Reversely, pushing the criterion to a higher value, such that it covers more of the unsure distribution, will improve the ability to predict “unsure” (thin red) and decrease the accuracy of predicting “sure” (thick red). **(C)** Average prediction accuracy in each subject. The criterion value was set to 0.55 [the maximum average prediction accuracy taken from the blue curve in **(B)**]. The accuracy using the same classifier when the sure and unsure class labels are drawn randomly (and so when the relationship between the neural signal and confidence is distorted) falls to chance (50%, range 49.9–50.1%; see magenta bars). The filled blue bars represent values that are significantly (*p* < 0.05) higher than the chance accuracy of 50% (Section 2).

Figure 5B shows that the confidence-related neural effect substantially precedes a button press choice. This finding leads to the question whether the signal could be used to predict, in each trial, and using neural signals collected prior to the button press, whether a subject is sure or unsure to choose the button press. To test this possibility, we averaged the alpha power over all channels in the period of the significance of the effect of confidence (bar below the abscissa in Figure 3C) for all training trials of a given subject. The averaging over all channels slightly improved the prediction because the effects of the choice confidence were, to a reduced degree, observed also at other channels. The choice confidence separates the resulting neural values into two (sure and unsure) distributions (Figure 8A). These distributions are used to set a prediction criterion according to the experimenter's demands on the level of type I and type II misclassification errors. A new, independent test-set (Section 2) signal value is then compared to the criterion. The classifier predicts that a subject is going to be sure (unsure) to choose the button press if that signal value is higher (lower) than the criterion.

We assessed the accuracy of the predictions from the neural signals against the confidence levels indicated by each subject on each trial. Validating the predictions on new, independent test-set neural signals ensures that the same level of prediction accuracy could be achieved in real time settings.

The average prediction accuracy as a function of the criterion is given in Figure 8B. When the criterion moves to the median of the distribution of the neural signals representing sure choices (criterion = 1.0), our ability to predict “unsure” should be high, and our ability to predict “sure” should converge to chance (50%). This is indeed what we observe (Figure 8B). The reverse pattern holds when the criterion is aligned with the median representing unsure choices (criterion = 0.0). Thus, the criterion can be chosen by the experimenter to meet particular demands on type I and type II error. We averaged the two accuracy measures (blue line in Figure 8B) to assign an equal weight to the trials on which a subject is sure and unsure. The maximum average accuracy of 60% is achieved midway between the medians of the two distributions—at the criterion value of 0.55.

The accuracy of the prediction of choice confidence for this criterion value is given separately for each subject in Figure 8C. The figure demonstrates that this simple neurally-informed procedure provides a substantial (up to 66% in one subject, 60% on average), consistent (all subjects greater than 50%) and significant (7 out of 10 subjects) ability to predict a subject's confidence in an upcoming button press. The accuracy converges to chance level (50%) when the relationship between the neural signals and the confidence labels is distorted (magenta bars aligning with the 50% value in Figure 8C, Section 2).

**Figure 9:**
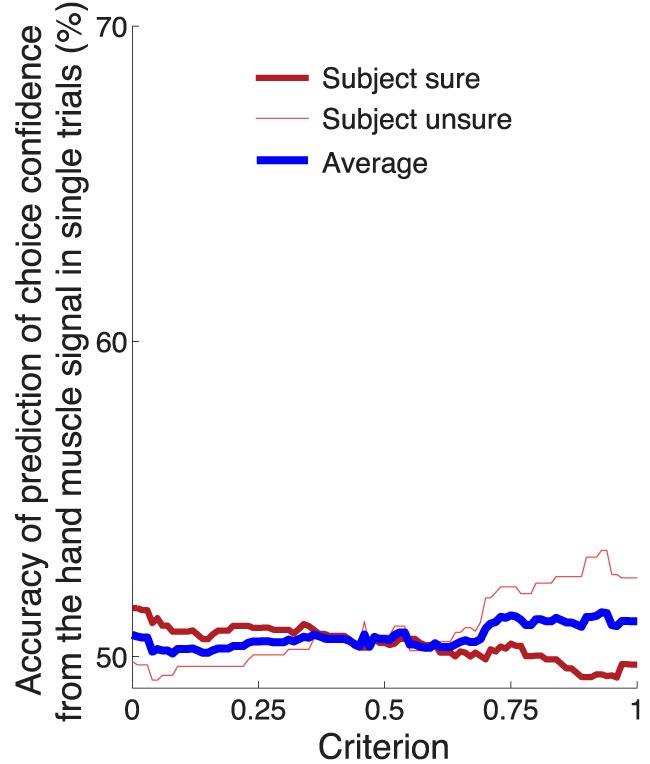
**Prediction of the confidence in a button press choice from forearm muscle activity**. Same analysis and format as in Figure 8B, using the EMG activity of flexor carpi radialis.

In comparison to the neural signal, the activity of the forearm muscles provided minimal information about the subjects' confidence to choose the button press (Figure 5C). We nonetheless investigated whether it is possible to infer the confidence in choosing the button press from the EMG activity of the forearm muscle flexor carpi radialis. Using this signal, we evaluated the prediction accuracy in the same way and over the same temporal window as with the neural signal (Figure 8). The result is shown in Figure 9. The maximum average prediction accuracy using the muscle signal is 51.5% (chance is 50%) and is achieved for the criterion value of 0.94. Thus, the ability to predict a subjects' confidence in an ensuing button press choice is contingent upon an access to a signal of the central nervous system.

Finally, we asked whether the alpha power contributes to the choice confidence with information beyond that provided by the other considered variables. To do so, we included the alpha power, the DV, RT, and the two movement-related variables as regressors on the choice confidence (Equation 4). We made sure that the confidence-related effects are not due to only a small subset of subjects, despite the finding that the effect is prevalent in most subjects (Figure 8C). To make sure that the effects are generalizable over a subject population, we considered each subject as a random effect influence on each factor (Equation 4). In this analysis, the alpha power was again averaged over all channels. Similar results were obtained when signals were measured only at CP3 or P3.

**Figure 10:**
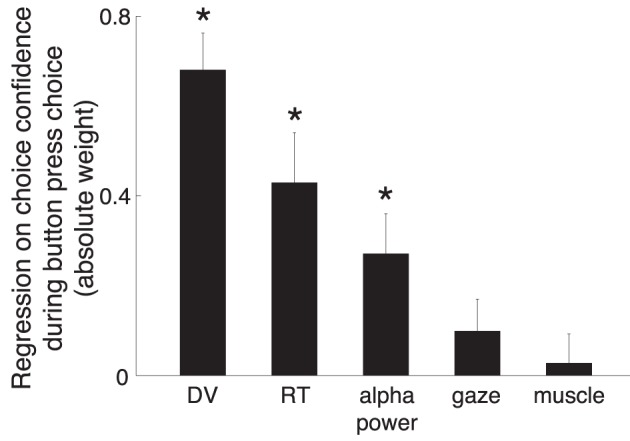
**The prediction of subjects' confidence from the individual factors**. Linear model (Equation 4) in which the individual factors (Section 2) are regressed on the choice confidence, during all button press choices. To ensure generalizability, the linear model considered each subject as a random effect influence on each factor. The weights were normalized (Section 2) so that a comparison of the weight magnitudes is fair. The error bar denote the SEM of each weight. For the purpose of visual comparison, the figure shows the absolute values of the weights. The text gives the actual values. ^*^p < 0.01.

Figure 10 shows that the modeled DV was the most informative factor in determining how confident a subject was going to be in choosing a button press (weight equal to 0.68, weight significance *p* < 0.001). This is not surprising—the DV reflects the amount of sensory evidence available to the subject, as does the confidence. The analysis shows that a knowledge of the RT is also helpful in determining a subjects' confidence [weight −0.43 (the more confident, the shorter the RT), *p* < 0.001]. The alpha power contributed with substantial additional information (weight −0.27, *p* = 0.0022). Subjects' gaze position or hand EMG activity did not (*p* > 0.16). Thus, from a perspective of a judge determining a subject's confidence, even if the DV and RT were available to the judge, the alpha power contributes substantial additional information in determining the subject's confidence.

## 4. Discussion

Many previous studies in animals and several recent studies in humans have shown that neural signals in several regions of the brain are modulated by decision-related variables (DV) in perceptual decision tasks (Shadlen and Newsome, 2001; Heekeren et al., 2004, 2008; Gold and Shadlen, 2007; Tosoni et al., 2008; Wang, 2008; Andersen and Cui, 2009; Ho et al., 2009; Kable and Glimcher, 2009; Ratcliff et al., 2009; Wunderlich et al., 2009; O'Connell et al., 2012; Kubanek et al., 2013). More recently, studies in animals demonstrate that several regions of the brain encode variables related to an animal's confidence in a choice, and that those signals might be distinct from those encoding the DVs (Kepecs et al., 2008; Kiani and Shadlen, 2009; Middlebrooks and Sommer, 2012; Komura et al., 2013). Here we extended these findings by investigating whether and how a confidence-related variable is encoded in human cortical activity in a perceptual decision task. We found that the degree of a subject's confidence in committing to a button press choice is strongly reflected in cortical activity in the alpha band.

The finding of a modulation of cortical signals by a person's confidence in an forthcoming choice is novel. Previous electrophysiological studies in humans focused on the investigation of the effects of confidence or awareness on the error-related negativity signal (ERN) that is specifically observed following an error (Hewig et al., 2011; Shalgi and Deouell, 2012), but not on the effect of confidence in an ensuing choice.

A previous study in monkeys (Kiani and Shadlen, 2009) suggested that the choice confidence may modulate neural signals independently of a DV, even though both the choice confidence and the DV are based on the amount of information in the stimulus (Vickers, 1979; Koriat et al., 1980; Vickers and Packer, 1982; Baranski and Petrusic, 1994, 1998; Koriat, 2011). The high temporal resolution of the EEG allowed us to investigate the contributions of the choice confidence and the DV to the alpha activity. We found that the choice confidence had an independent leverage on the neural signal, and exhibited somewhat different dynamics than the effect of the DV (Figure 3C). Furthermore, the neural signal encoded to the choice confidence even when the DV was taken into account (Figure 10). Although this result may suggest (as it did in Kiani and Shadlen, 2009) that the choice confidence and the DV may be partially independent phenomena, this interpretation must be taken with care. For instance, it is possible that other forms of DVs, or forms other than the linear models to test for independence between the variables, may lead to different interpretations.

A more general criticism of studies investigating neural representations of choice confidence has been whether the subjective concept of confidence is a more useful descriptor than objective, quantitative variables, such as the decision-related variables (DVs). In particular, deviating from an objective description of a degree of commitment in a choice (Shadlen and Newsome, 2001; Heekeren et al., 2004, 2008; Gold and Shadlen, 2007; Tosoni et al., 2008; Wang, 2008; Andersen and Cui, 2009; Ho et al., 2009; Kable and Glimcher, 2009; Ratcliff et al., 2009; Wunderlich et al., 2009; O'Connell et al., 2012; Kubanek et al., 2013) incurs several difficulties. Perhaps the most notable is that the concept of confidence is intertwined with many other concepts. When subjects obtain more evidence, they are typically more confident to make a choice, are faster to make a choice, and commit less errors (McDougall, 1921; Festinger, 1943; Vickers, 1979; Koriat et al., 1980; Vickers and Packer, 1982; Gigerenzer et al., 1991; Baranski and Petrusic, 1994, 1998; Koriat, 2011; Yeung and Summerfield, 2012). Thus, confidence is confounded by sensory evidence, RT, choice correctness or accuracy, and likely many other variables. It is arguable to what extent researchers can control for such confounds when investigating the neural representations of the choice confidence. Moreover, it is arguable whether making such a distinction is even useful given the inherent natural relationships between these variables during decision-making. Another point to keep in mind is that subjective confidence judgements are likely affected by many influences that are difficult to control for, such as a subject's previous judgment or a subject's overall level of confidence.

However, there are three points of merit in establishing that a signal is a neural correlate of choice confidence.

First, such a signal can provide a window into a subject's commitment to make a choice (Figure 5B) without having to generate a model of a DV (Shadlen and Newsome, 2001; Heekeren et al., 2004, 2008; Gold and Shadlen, 2007; Tosoni et al., 2008; Ho et al., 2009; Ratcliff et al., 2009; Wunderlich et al., 2009). This may be beneficial in situations in which experts make complex decisions that cannot be readily modeled by an experimenter.

Second, even when a DV can be successfully defined by an experimenter, our data show that the neural signal can be used to provide additional independent information about a subject's choice confidence (Figure 10).

Third, we found that choice confidence modulates the alpha-band signal strongly, and the modulation substantially precedes the time at which a subject commits to a button press. We found that the level of a subject's confidence could be read out, in single trials, from the brain signals recorded prior to the behavioral outcome (Figure 8). Given a recording during the stimulus period on a given trial, a simple thresholding classifier trained on an independent data set predicted whether a subject was going to be sure or unsure in the button press choice with an average accuracy of 60%. As a control, the choice confidence could not be inferred from peripheral muscle signals (Figures 5C, 9). The finding that a non-invasively acquired neural signal predicts the confidence in an impending button press choice could be applied to infer how confident an operator is going to be in committing to an action. Of course, to arrive to such an application, the approach would have to be greatly refined in the future.

We designed the task to feature two distinct response effectors, which allowed us to distinguish whether an effect is specific to a particular movement system. This helped establish that the alpha-band signal is modulated by the choice confidence specifically during button press choices. The specificity of the choice confidence effect to button press choices excludes the possibility that the effect could be due to an artifactual influence (e.g., eye blinks), or due to a generic cognitive process, such as attention (Gottlieb, 2007; Peck et al., 2009), reward expectation (Kable and Glimcher, 2009), motivation (Roesch and Olson, 2004), or task difficulty (Chen et al., 2008). Furthermore, this finding suggests that this neural effect specifically develops in circuits that are tied to the somatomotor system. This raises the question whether the effects can be detected in other systems as well. In this regard, interestingly, the dynamics of the confidence-related neural effects reported here are similar to those observed in the monkey oculomotor system (Kiani and Shadlen, 2009). In both studies, a modulation due to choice confidence is apparent during an early portion of a trial, while a stimulus is still present [about 400 ms relative to stimulus onset in our study compared to about 300 ms—Figure 2D (Kiani and Shadlen, 2009)]. In both studies, the effects vanish around the time of a movement. Thus, the dynamics of neural representations of a developing commitment (confidence) may be similar across effector systems. Notably, the finding that the alpha power effect of choice confidence reported here is specific to manual choices does not preclude the possibility that there are other signals that encode a subjects' confidence in a general, effector-independent manner. Within our paradigm, we did not observe robust confidence-related signals during saccade choices, but this remains to be tested in the future, potentially using a more sensitive recording approach.

Our finding that the neural effect of the choice confidence or DV was specific to button press choices could reflect an effect of motor planning (Bestmann et al., 2008). There are three indications that the effect is not of a purely motor nature. First, the effect of the choice confidence is observed even after accounting for movement plans (button presses), i.e., when only trials that resulted in a button press were considered in the analysis. Second, the effects of the choice confidence and the DV during button press choices are significant already early in the trial (350–400 ms following stimulus onset Figure 3B), which on average is more than a second before a subject performs a button press. Third, in contrast to the relatively early neural effect, the EMG activity of the forearm muscles—which directly reflects motor planning and preparation—was not observed until shortly prior to a button press (Figure 5). These findings suggest that the alpha-band neural effect reflects a *higher-order* decision-related process that is specifically tied to the somatomotor system. This is supported by recent findings that alpha-band signals in perceptual decision tasks reflect decision- and confidence-related variables already before the onset of a perceptual stimulus (de Lange et al., 2013; Baumgarten et al., 2014; Lou et al., 2014).

An action is typically executed when a subject is sure about performing that action, and withheld when the subject is unsure (Kiani and Shadlen, 2009; Komura et al., 2013). Thus, the confidence in choosing an action can be thought of as a cognitive variable that can act on a gate that either gives rise to or inhibits an action. It has been proposed that the alpha rhythm, the signal identified in the present study, reflects an operation of a gate that gives rise to or inhibits an action (Lopes da Silva, 1991; Mink, 1996; Leblois et al., 2006). In particular, the gating function is implemented by the thalamo-cortical neuronal interaction, which appears to modulate the alpha rhythm in regard to generation or inhibition of an action (Steriade and Llinás, 1988; Pfurtscheller et al., 1996; Kubanek et al., 2013). Hence, the alpha rhythm may provide a unique view on the operation of this subcortical cognitive gate (Saalmann and Kastner, 2009; Saalmann et al., 2012), and thereby reveal the state of a subject's confidence in an upcoming somatomotor action. This hypothesis provides a set of testable predictions that could be investigated in invasive studies in the future.

In summary, cortical alpha activity reflects a variable related to a degree of a subject's confidence in an upcoming button press choice. This effect is marked and independent of movement-related variables controlled for in this study. This signal provides a probe into the dynamics of a subject's deliberation in a forming decision and enables a prediction of the level of confidence associated with a forthcoming button press choice.

### Conflict of interest statement

The authors declare that the research was conducted in the absence of any commercial or financial relationships that could be construed as a potential conflict of interest.
